# Cerebellar Infarction Diagnosed by the 'Hyperdense Sign' on Plain CT

**DOI:** 10.7759/cureus.86006

**Published:** 2025-06-14

**Authors:** Shunsuke Nakamura, Natsuyo Shinohara, Shigeaki Inoue

**Affiliations:** 1 Department of Emergency and Critical Care Medicine, Wakayama Medical University, Wakayama, JPN

**Keywords:** cerebellar infarction non-contrast ct, hyperdense sign, posterior circulation, stroke, vertebral artery dissection

## Abstract

We hereby present a case of thrombus or possible vertebral artery dissection (VAD), but no confirmatory evidence in a young adult male, diagnosed using an unusual but pivotal imaging clue, the "dense vertebral artery sign" observed on non-contrast computed tomography (CT). This finding led to the initial suspicion and subsequent confirmation of VAD. This report underscores the diagnostic value of subtle vascular signs on plain CT imaging that are frequently overlooked but can result in prompt and life-saving intervention. This report is of particular significance in emergency settings, where advanced imaging modalities such as MRI and CT angiography may not be readily available.

## Introduction

Vertebral artery dissection (VAD) is a primary cause of ischemic stroke in younger adults, particularly those under the age of 45. The prevalence of VAD is likely underestimated due to its frequently non-specific clinical presentation. Common symptoms include sudden-onset dizziness, neck pain, headache, and focal neurological deficits, which may mimic other less severe conditions [1,2]. Consequently, delayed diagnoses are not infrequent, and prompt recognition is imperative for effective management.

Although magnetic resonance angiography (MRA) and computed tomography angiography (CTA) are widely regarded as the prevailing standards for vascular imaging, these modalities may not be readily accessible during the initial evaluation. In such settings, findings on non-contrast CT, specifically the "hyperdense vertebral artery sign," can offer crucial diagnostic information and facilitate rapid decision-making [3]. While the hyperdense middle cerebral artery (MCA) sign is the focus of more research, the hyperdense vertebral artery sign merits greater attention in emergency neuroimaging protocols.

## Case presentation

A 43-year-old male with no previous medical history of note presented to the emergency department with a sudden onset of vertigo, slurred speech, and left-sided motor weakness. The patient denied recent trauma, infection, or prior neurological episodes. A physical examination of the patient revealed that he was alert and oriented, with stable vital signs. A neurological assessment revealed dysarthria, mild left-sided limb ataxia, and reduced coordination, suggesting cerebellar involvement.

**Figure 1:**
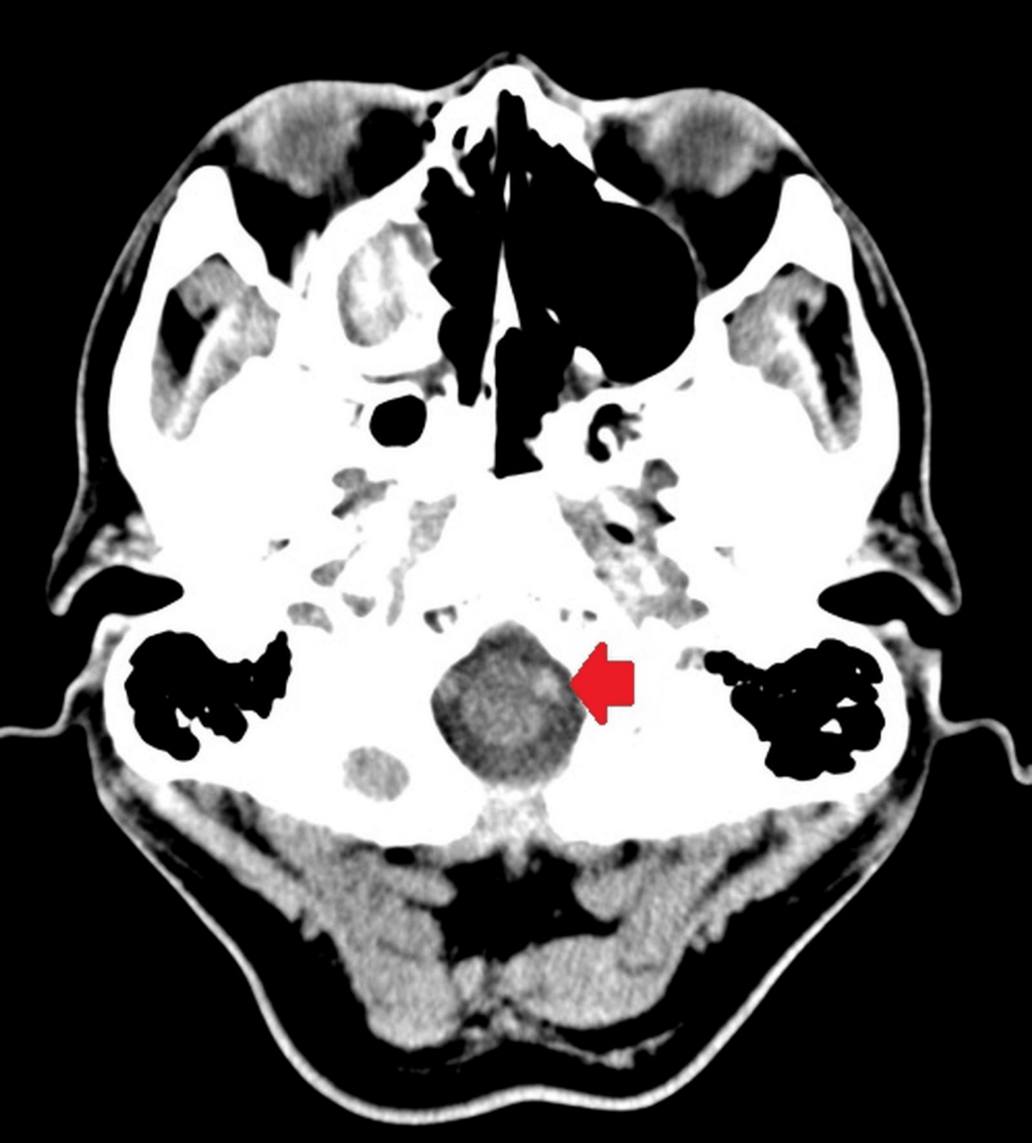
A non-contrast CT scan shows a hyperdense appearance of the left vertebral artery, indicating a hyperacute cerebral infarct image in the vertebral artery region (red arrow), suggestive of thrombus or dissection and hyperacute cerebral infarction.

Consequently, an immediate non-contrast head CT was performed to rule out the possibility of a hemorrhagic stroke. Although no intracranial bleeding was detected, a focal area of increased attenuation was noted along the expected course of the left vertebral artery, consistent with the hyperdense vertebral artery sign (Figure 1).

**Figure 2:**
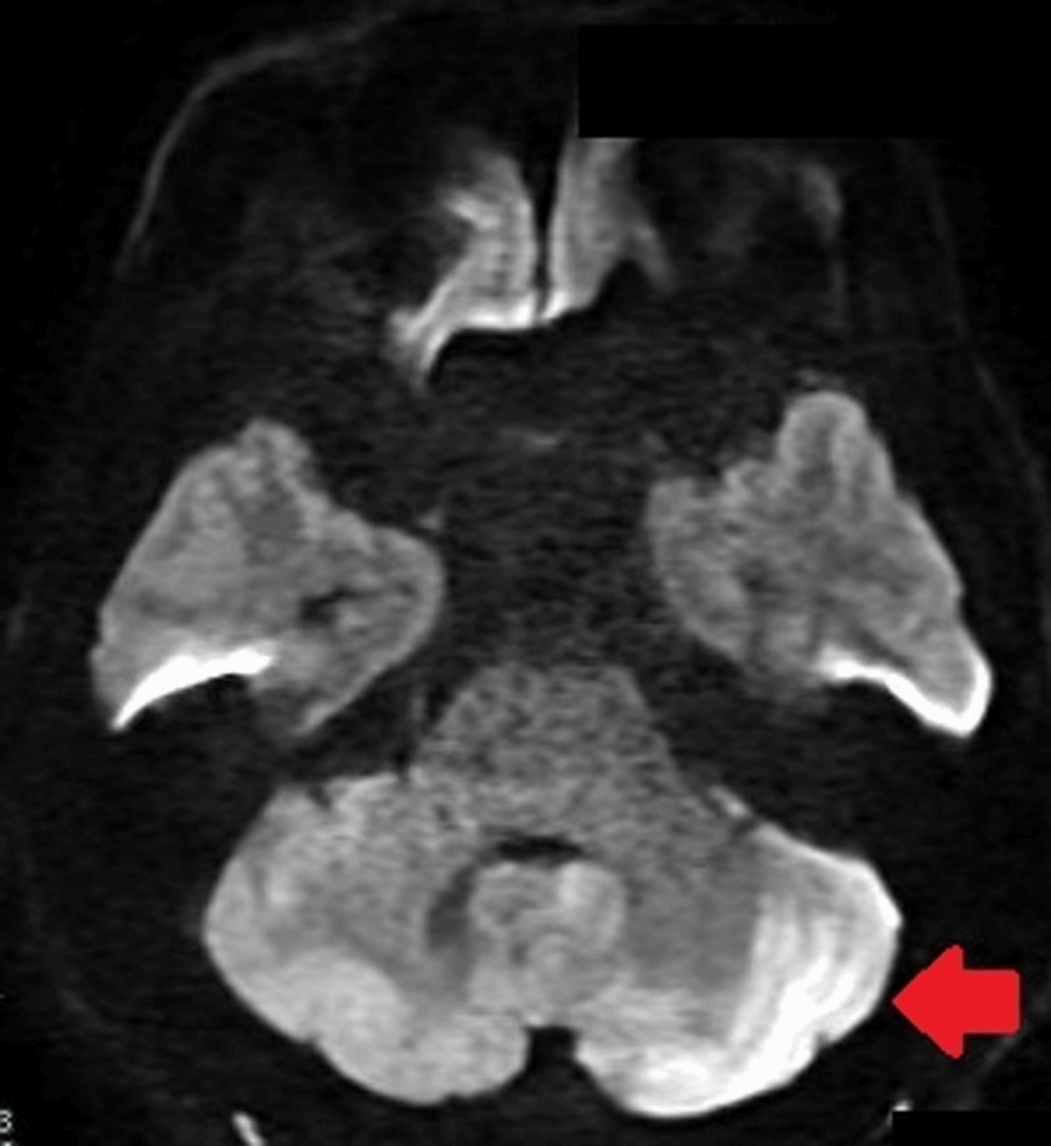
MRI DWI shows a hyperacute cerebral infarct in the posterior inferior cerebellar artery region (red arrow), suggestive of thrombus or dissection and hyperacute cerebral infarction. MRI: magnetic resonance imaging; DWI: diffusion-weighted imaging

Consequently, further evaluation was conducted using MRI and MRA, which confirmed the presence of an acute infarction in the territory of the left posterior inferior cerebellar artery (PICA) and occlusion of the left vertebral artery. This substantiated the diagnosis of thrombus or possible VAD, but no confirmatory evidence (Figure 2).

The patient was transferred to a tertiary stroke center for consideration of endovascular treatment and antithrombotic therapy.

## Discussion

This case demonstrates the clinical utility of the hyperdense vertebral artery sign as an early radiological marker of thrombus or possible dissection, but no confirmatory evidence. While this sign is not pathognomonic, its presence in the appropriate clinical context, such as posterior circulation stroke symptoms in a young adult, should prompt immediate further investigation.

The hyperdense vertebral artery sign on non-contrast CT is considered analogous to the better-known hyperdense MCA sign [4,5]. It signifies the presence of intraluminal thrombus or slow-flowing blood, which manifests as increased attenuation within the vessel lumen.

The following text is intended to provide a comprehensive overview of the subject matter. Gottesman et al. conducted a systematic review and found that, although the hyperdense sign is reported less frequently in vertebral arteries than in the MCA, it possesses significant diagnostic implications when present [3].

Non-contrast CT is frequently the primary imaging modality utilized in emergency settings due to its accessibility and expeditious nature. Although MRA and CTA are the gold standard modalities for detecting vascular abnormalities (such as VAD), they may not always be readily accessible during the initial assessment. In such circumstances, non-contrast CT findings can serve as critical early indicators. In addition to the famous MCA sign, several others that can be noted with simple CT have been reported [6]. The presence of subtle vascular hyperdensity on non-contrast CT scans should prompt targeted vascular imaging, particularly in young patients devoid of vascular risk factors.

The prevalence of VAD among young stroke patients is noteworthy. The prevalence of this condition has been documented to range from 10 to 20% among individuals under the age of 45, thus underscoring the critical importance of timely identification [7]. Nonetheless, the clinical presentation is frequently non-specific, encompassing symptoms such as headache, vertigo, and cerebellar signs.

This case underscores the critical importance of radiological education. It is imperative that emergency physicians and radiologists receive training to recognize early signs of arterial dissection on plain CT. The extant literature emphasizes that familiarity with vascular signs, including the hyperdense vertebral artery sign, can facilitate the expeditious initiation of appropriate management.

Furthermore, early recognition and intervention may prevent long-term neurological sequelae. Patients with early VAD diagnoses could exhibit superior outcomes in comparison with those whose diagnoses were postponed. Consequently, it is imperative to underscore the clinical significance of identifying even the most subtle indications on initial imaging.

In summary, while advanced imaging remains the diagnostic gold standard, this case underscores the enduring value of plain CT when interpreted with appropriate clinical suspicion and radiologic awareness. The hyperdense vertebral artery sign is an uncommon phenomenon that is nevertheless of significant diagnostic consequence.

## Conclusions

VAD is a critical yet underdiagnosed cause of posterior circulation stroke, especially in young adults. This case highlights the importance of maintaining a high level of clinical suspicion and conducting a meticulous image review during the initial workup. The hyperdense vertebral artery sign could suggest VAD as an early indicator on plain CT.

Emergency clinicians should be aware of this sign and consider further vascular imaging promptly when encountered, especially in patients with compatible neurological presentations. Early recognition facilitates timely treatment, reduces the risk of recurrent stroke, and improves overall patient outcomes.
